# Comparative genomics identifies potential virulence factors in *Clostridium tertium* and *C. paraputrificum*

**DOI:** 10.1080/21505594.2019.1637699

**Published:** 2019-07-13

**Authors:** Marina Muñoz, Daniel Restrepo-Montoya, Nitin Kumar, Gregorio Iraola, Giovanny Herrera, Dora I. Ríos-Chaparro, Diana Díaz-Arévalo, Manuel A. Patarroyo, Trevor D. Lawley, Juan David Ramírez

**Affiliations:** a Grupo de Investigaciones Microbiológicas – UR (GIMUR), Programa de Biología, Facultad de Ciencias Naturales y Matemáticas, Universidad del Rosario, Bogotá, Colombia; b Posgrado Interfacultades, Doctorado en Biotecnología, Facultad de Ciencias, Universidad Nacional de Colombia, Bogotá, Colombia; c Genomics and Bioinformatics Program, North Dakota State University, Fargo, ND, USA; d Host–Microbiota Interactions Laboratory, Wellcome Trust Sanger Institute, Hinxton, UK; e Microbial Genomics Laboratory, Institut Pasteur Montevideo, Montevideo, Uruguay; f Center for Integrative Biology, Universidad Mayor, Santiago de Chile, Chile; g Molecular Biology and Immunology Department, Fundación Instituto de Inmunología de Colombia (FIDIC), Bogotá, Colombia; h Faculty of Animal Sciences, Universidad de Ciencias Aplicadas y Ambientales (UDCA), Bogotá, Colombia; i School of Medicine and Health Sciences, Universidad del Rosario, Bogotá, Colombia

**Keywords:** Clostridium tertium, Clostridium paraputrificum, Clostridial species, genetic diversity, virulence factors

## Abstract

Some well-known Clostridiales species such as *Clostridium difficile* and *C. perfringens* are agents of high impact diseases worldwide. Nevertheless, other foreseen Clostridiales species have recently emerged such as *Clostridium tertium* and *C. paraputrificum*. Three fecal isolates were identified as *Clostridium tertium* (Gcol.A2 and Gcol.A43) and *C. paraputrificum* (Gcol.A11) during public health screening for *C. difficile* infections in Colombia. *C. paraputrificum* genomes were highly diverse and contained large numbers of accessory genes. Genetic diversity and accessory gene percentage were lower among the *C. tertium* genomes than in the *C. paraputrificum* genomes. *C. difficile tcdA* and *tcdB* toxins encoding homologous sequences and other potential virulence factors were also identified. *EndoA* interferase, a toxic component of the type II toxin-antitoxin system, was found among the *C. tertium* genomes. *toxA* was the only toxin encoding gene detected in Gcol.A43, the Colombian isolate with an experimentally-determined high cytotoxic effect. Gcol.A2 and Gcol.A43 had higher sporulation efficiencies than Gcol.A11 (84.5%, 83.8% and 57.0%, respectively), as supported by the greater number of proteins associated with sporulation pathways in the *C. tertium* genomes compared with the *C. paraputrificum* genomes (33.3 and 28.4 on average, respectively). This work allowed complete genome description of two clostridiales species revealing high levels of intra-taxa diversity, accessory genomes containing virulence-factors encoding genes (especially in *C. paraputrificum*), with proteins involved in sporulation processes more highly represented in *C. tertium*. These finding suggest the need to advance in the study of those species with potential importance at public health level.

## Introduction

The Clostridiales bacterial order contains Gram-positive and Gram-negative members that display a wide range of morphological features, metabolic variations, different spore characteristics, and ecology patterns. Some Clostridiales species are beneficial to their human and animal hosts [1], but others are pathogenic and potentially hazardous to them [1,2]. At least 15 families fall within the Clostridiales order [3], and the Clostridiaceae family includes important opportunistic pathogens such as *Clostridium botulinum* [4], *C. perfringens* [5] and *C. tetani* [6]. *C. difficile*, an additional Clostridiales species of public health relevance [7], belongs to the Peptostreptococcaceae family [8].

Recently, thanks to the increased use of genomic epidemiology and metagenomics technologies [9], many Clostridiaceae species have been identified and/or taxonomically assigned [3] using classical 16S RNA sequence analyses. However, the limited resolution of this molecular marker has led to some species remaining unclassified within the Clostridiales order, as can be demonstrated by searching the NCBI taxonomy browser using the keyword “Clostridiaceae” (https://www.ncbi.nlm.nih.gov/Taxonomy/Browser/wwwtax.cgi) [10,11]. Information is also lacking on the biological features of the recently identified Clostridiales species, despite most of them presenting a public health risk because of the severe infections they cause, their increased association with global disease outbreaks, and the emergence of hypervirulent strains that may also have acquired antibiotic resistance [12].

Two Clostridiales species, namely, *C. paraputrificum* and *C. tertium*, are described as uncommon pathogens in humans, despite being linked with various cases of severe disease in people infected with them. Colonization by *C. paraputrificum* is mainly associated with myonecrosis and bacteremia in humans although a wide range of invasive infections can also occur [13], and this pathogen was recently found to be the fortuitous cause of necrotizing cellulitis of the abdominal wall in one individual [14]. *C. tertium* is associated with bacteremia cases [15] and septic shock [16], but it mainly infects immunocompromised [17] and neutropenic patients [18]. This species has recently been identified as a causal agent of necrotizing fasciitis and gangrene [19], and is also known to cause various pathologies in non-neutropenic patients [20].

Although the pathogenic effects of most Clostridiales species are related to their toxin producing abilities [21], this ability also shows species variation. For *C. paraputrificum*, the pathogenic effect on its hosts has been historically attributed to the action of chitinases [22], whereas for *C. tertium*, sialidase production is more important [20]. However, misidentification during routine microbiological testing means that the virulence factors from these species have not been studied exhaustively [23]. The draft genomes for *C. tertium* and *C. paraputrificum* were recently published [24,25], where exploratory phylogenetic analysis (to differentiate genomes from their closest relatives) and virulence-related factors and antimicrobial resistance genes were predicted by *in silico* analysis exclusively. However, no detailed experimental characterization on the virulence factors encoded within these genomes has been conducted to date.

Therefore, the present study aimed to obtain a detailed description of the *C. paraputrificum* and *C. tertium* genome assemblies from the isolates accidentally recovered during screening for *C. difficile* infections in stool samples from human adults with diarrhea in Colombia. The taxonomic allocation, intra-taxa genomic variations and potential virulence factors were also analyzed from whole genomes using a range of approaches. Considering the relevance of the virulence factors found, a subsequent phenotypic verification of their cytopathic potential and sporulation efficiency of these isolates was developed. This study represents a baseline about the virulence factors transported by these species, mainly *C. paraputrificum*, so that in the future it could favor the management schemes of infected patients.

## Material and methods

### Clinical isolates

Clinical isolates were obtained from stool samples from adult patients with diarrhea via the CDI detection scheme. The implemented scheme was directed at detecting healthcare facility-onset (HCFO) and community onset (CO) CDIs under the project framework “Characterization of *Clostridium difficile* in Bogotá, Colombia” which involved two Colombian healthcare centers (Méderi and Shaio Clinic Foundation). The inclusion criteria, methodology used and main findings for the sample set are described in a previous study by our group [26]. Gcol.A2 and Gcol.A11 were isolated from the HCFO samples obtained from patients with previous histories of treatment with multiple antibiotics who were transferred to the intensive care unit. The antibiotics included albendazole, ivermectin and ertapenem for Gcol.A2, and meropenem, metronidazole and teclozan for Gcol.A11. The third isolate (Gcol.A43) was recovered from a CO sample. Information on the antibiotic consumption history is not available for the CO patient (see “Ethics approval and consent to participate” section). The complete clinical information on the patients is available in Table S1.

One approach used to detect CDIs is *in vitro* culturing, whereby an initial fraction from each fecal sample (~200μL) is quickly extended by streaking it onto selective chromogenic medium (chromID *C. difficile* agar; bioMérieux SA, Craponne, France) followed by incubation for 48 h at 37°C under anaerobic conditions using the GasPak EZ Anaerobe container system (Becton Dickinson, Franklin Lakes, NJ, USA). Colonies with the macroscopic morphologies described by the manufacturer (grey to black with irregular or smooth borders) were screened by spreading them onto Trypticase ™ I Agar (TSA) containing 5% sheep blood (Becton Dickinson), to verify their macro and microscopic morphologies through routine Gram staining interpretation, after an incubation period under the aforementioned conditions. The biomass of all the colonies corresponding to Gram-positive bacilli (occasionally sporulated) increased when the conditions described previously for colony screening were used.

The cell biomass was recovered for three purposes: 1) for cryopreservation via resuspension in 500 μL of Oxoid nutrient broth (Thermo Fisher Scientific, MA, USA), containing 20% (v/v) glycerol (Thermo Fisher Scientific) with subsequent storage at −80°C; 2) as a source material for DNA extraction via recovery in 300 μL of 1X phosphate-buffered saline (PBS), followed by storage at −20°C until processing. In both cases, the bacterial biomass was recovered from the TSA medium at an optical density (OD) of up to 600 nm (OD_600_), which is equivalent to 4 × 10^7^ cells per mL; 3) as a sample source for further microbiological testing, when a fuller description of the established isolate was required.

### Ethics approval and consent to participate

The initial study aimed to detect *C. difficile* infections in fecal samples from patients with diarrhea and was approved by the Universidad del Rosario’s Research Ethics Committee (Approval Act No. 290, 27 July 2015). In addition, an addendum was approved, which authorized the additional use of samples for research purposes aimed at the description and characterization of any microorganisms present in the human gastrointestinal tract (Approval Act No. 312 28 April 2016). This ethics committee approved the use of the microorganisms isolated from the patients. All patients included in this study agreed to participate and signed informed consent forms agreeing to their participation in the study.

### Preliminary descriptions of the clinical isolates

Phenotypic tests to presumptively identify and classify the clostridiales clinical isolates were conducted. The third portion of bacterial biomass from each established isolate (described in the previous section) was used to develop the following basic microbiological tests: malachite green spore morphology staining [27] with subsequent contrast of the vegetative cells using safranin staining; catalase assessment via hydrogen peroxide decomposition determination [28]; motility and urease assessments, using sulfide indole motility medium (Thermo Fisher Scientific); and glucose and lactose fermentation assessment using Oxoid Kligler iron agar (Thermo Fisher Scientific). All the microbiological tests were conducted in duplicate.

The preliminary descriptions of the isolates were further verified by determining the protein fingerprints of the whole cells using MALDI-TOF MS, and subsequent identification by database matching, as described previously [29].

### DNA extraction and whole genome sequencing

Bacterial pellets recovered in 1× PBS were subjected to DNA extraction using the Ultraclean BloodSpin DNA Isolation kit (MoBio Laboratories, Carlsbad, CA, USA), according to the manufacturer’s instructions. The extracted DNA was recovered in 100 μL of elution buffer and its quality verified by spectrophotometric quantification (NanoDrop2000, Thermo Scientific NanoDrop products) and agarose gel electrophoresis, followed by SYBR Safe staining of the gels (Invitrogen, Gaithersburg, MD, USA). The extracted DNA was used for whole genome sequencing commercially (Novogene Bioinformatics Technology Co., Ltd, Beijing, China), using the HiSeq X-TEN System (Illumina). The microbial mate-paired libraries constructed by end repair (insert size, 350-bp) were subjected to paired-end sequencing (read length, 2 × 150-bp). A preliminary data analysis was developed to obtain high-quality, clean data, whereby the paired reads were discarded when a read contained adapter contamination, a read contained uncertain nucleotides of more than 10%, or a read contained low quality nucleotides (base quality <5) of more than 50%. This standard has been previously approved for scientific research [30]. To guarantee data reliability, QC was performed during each step of the sequencing procedure.

### Data retrieval

Database searching was conducted to select a set of representative genomes for inter- and intra-species comparisons. For this, we accessed the bacterial genomics database website PATRIC (Pathosystems Resource Integration Center; https://www.patricbrc.org/) [31,32], the European Nucleotide Archive (ENA) [33] and the data resource at the National Center for Biotechnology Information (NCBI) [34], and the complete genome sequences acquired were downloaded in FASTA format. To obtain a wide range of data we used the following criteria: *Clostridiales, Clostridiaceae* and *Clostridium*. A QC procedure was conducted on all the downloaded genome sequences, using the GenomeQC_Filter_v1-5 script [35], which considers the following main parameters for a permitted genome: a maximum of 400 contigs per genome, a maximum genome size of 8 MB, with the presence of 16S ribosomal RNA gene sequences. Subsequently, alignment using SILVA Incremental Aligner (SINA) service (a program designed to align 16S genes using the SILVA database provided by SILVA rRNA project [36]) was conducted, which allows to evaluate the taxonomic assignment, together with the identification of potential contamination by determining the similarity percentage between the 16S sequences from the same genome. For the SINA Service [36] analysis, it was condidered 95.0% to be the minimum identity percentage for each query sequence and employs linked databases (SILVA, RDP, greengenes, LTP and EMLB) all of which are included in The SILVA ribosomal RNA gene database project [36]. The *B. coagulans* (strain HM-08) genome acted as outgroup.

### Taxonomic placement for *C. tertium* and *C. paraputrificum*

Taxxo v1.0, a package in R [37,38] designed to elucidate the taxonomic classification of prokaryotic species from complete genome information, was used to taxonomically assign the isolates. First, we extracted the small subunit 16S rRNA gene sequences of the analyzed genomes using the *rrna* function included inside TAXXO v1.0. Then, a sequence similarity search was conducted using the BLAST algorithm [39] to identify 16S rRNA gene sequences of other clostridiales species that were included within the dataset analyzed for the phylogenetic reconstruction of this molecular marker. In parallel, the set of 16S rRNA gene sequences was aligned using SILVA Incremental Aligner (SINA) service [36] to predict the taxonomic classification of the genomes, considering the previously described parameters.

Secondly, high-resolution phylogenies were constructed on the downloaded data using the *uprot* function, which automatically identifies, extracts, concatenates and aligns sequences against a set of 40 universal single-copy gene-associated protein markers [40]. The *uprot* function was applied to the amino acid sequences obtained from the open reading frame reads predicted by Prodigal software [41], which included the prodigal function within Taxxo v1.0. This approach was selected to optimize the phylogenetic inferences, which currently represents the biggest challenge in the era of genome-scale datasets [42]. Approximate maximum-likelihood phylogenetic trees were generated from the amino acid sequence alignments obtained from *uprot* using FastTree under the Jukes-Cantor model of nucleotide evolution [43]. Bootstrap method (BT; with 1,000 replicates) was used for evaluating the nodes’ robustness [44], considering as well supported nodes, those with results ≥90.0%. The graph visualization of the phylogenetic trees was obtained from the Interactive Tree Of Life V3 (http://itol.embl.de) web-based tool [45]. The phylogeny obtained from the complete genome dataset that exceeded the advanced QC process described above was compared with the results obtained from the SINA Service to evaluate the usefulness for *Clostridiales* family and species groupings.

Subsequently, representative genomes for each Clostridiales family and the closest related species with target genomes were selected to establish a definitive genome dataset. A new phylogenetic reconstruction based on *uprot* was generated, followed by a detailed analysis of the phylogenetic relationships among the genomes following the same scheme, with the aim of identifying the taxonomic designation of target species and the most closely related species.

The phylogenetic signals identified by the Taxxo v1.0 package were compared against those using the classical approach to evaluate the phylogenetic relationships of the Clostridiales species based on their 16S rRNA sequences^3^. This involved taking the 16S rRNA gene sequence alignment extracted during the advanced QC process and using the same parameters on it that are described above.

### Assembly and annotation

The sequence reads were assembled *de novo* using an improved Illumina data pipeline for prokaryotes [46]. The sequence reads from each isolate were also used to create multiple assemblies with Velvet v1.2 [47] and VelvetOptimiser v2.2.5 (https://packages.debian.org/buster/velvetoptimiser). An assembly improvement step was applied to the assembly, and the best N50 and contigs were scaffolded using SSPACE [48] and the sequence gaps were filled by GapFiller [49]. Automated annotation was performed using underlying software of this pipeline is Prokka v1.13 [50], followed by a set of improvement steps was used, as follows: Infernal [51] was first run to predict RNA structures. Prodigal [41] is then run to predict proteins. Aragorn [52] was used to predict tRNAs and tmRNAs, and Rnammer [53] was used to predict ribosomal RNAs. The predicted genes were then annotated with data from databases searched in the following order: genus specific databases were generated by retrieving the annotation for all of the genomes for *Clostridium* genus from RefSeq [54]. The protein sequences were then merged using CD-hit [55] to produce a non-redundant blast protein database. Next UniprotKB/SwissProt [56] was searched, considering kingdom specific databases for Bacteria. Finally, each protein was then looked up against the HMM profiles from Clusters, Conserved domain database, Tigrfams, and Pfam. The software packages were developed by the Pathogen Informatics team at the Wellcome Trust Sanger Institute and are freely available to download from GitHub (https://github.com/sanger-pathogens/vr-codebase) under the GNU GPL 3 open source license. The circular genome visualization tool was developed in the CGview server [57], where the additional preliminary comparative genome analyses were conducted.

### Intra-taxa comparisons

A dataset including in the intra-taxa analyzes was generated from the final phylogeny using *uprot*. The genomes that consistently grouped within the same species cluster in the analysis were selected and subjected to a species delimitation step using ANI, which is available as an *anib* function within the Taxxo v1.0 package. Genomes with ANI values above 95.0 were considered to belong to the same species [58].

A pairwise comparison to identify intra-taxa differences in the whole genome data was carried out using Circoletto, a visualizing sequence similarity tool within Circos [59]. The genetic distance measured in terms of SNPs was determined for each comparison using NUCmer (NUCleotide MUMmer) version 3.1, a MUMMER tool [60]. All the genomes included in the dataset defined for each species evaluated were submitted to the aforementioned annotation pipeline. The resultant .gff files were used to define the pan genome with Roary [61], that extracts the coding regions and performs a theoretical translation of aminoacids, then iteratively pre-clustered with CD-HIT^82^ and finally make comparisons with BLASTP (considering as threshold a 95% percentage sequence identity). An order of occurrence is generated, in order to define core genome (defined as a gene being in at least 99% of samples) and accessory genome (genes in varying combinations). A phylogenetic reconstruction was created from the Multi-FASTA alignment of the core conserved genes (According to the parameters decribed for the methodology of “Taxonomic placement”), to determine the relationships between the isolates of interest. This approach was selected because it is recognized as having an “increased accuracy from the context provided by conserved gene neighborhood information”. Then, it was carried out to identify the distance between the genomes analyzed from the genome data of the same species. At present, this type of analysis is recognized as the most robust source of data for the clustering of isolates as reported elsewhere [62]. The core-genome tree generated was compared with a matrix in which the core and accessory genes where either present or absent, graphed using roary_plots.py Python script [https://github.com/sanger-pathogens/Roary] [63]. The NAG and NUG values were determined for each genome.

### Virulence factor identification

We used three different approaches to identify the molecular markers that potentially confer antibiotic resistance or represent OVFs in the isolates. One of these approaches involved screening the assemblies with the ABRicate program, which uses CARD, a comprehensive antibiotic resistance gene database [64], as well as Resfinder, ARG-ANNOT, NCBI BARRGD, NCBI, EcOH, PlasmidFinder and VFDB databases [65]. The second one involved the use of Ariba, a highly efficient tool aimed at detecting loci and mutations related to virulence factors or antibiotic resistance markers directly from the reads obtained from the sequencing process [66]. The third one, corresponded to a manual search of annotation outputs, followed by a comparison of the sequences of each candidate marker, against the information available in databases, using BLAST algorithm [39].

### Sporulation proteins detection

Proteins involved in the sporulation process were predicted by comparing a set of reference proteins known to be involved in sporulation pathways, with each of the genomes of interest acting as the query sequences. The reference set of proteins were those reported for *C. botulinum* Ba4 657 uid59173, which are available in the *Clostridium* Information Database, ClosinDB [67]. This reference genome was selected because *C. botulinum* is the only species genetically related to *C. paraputrificum* and *C. tertium* for which there is a reference genome, according to the phylogenetic analyzes conducted in this study. Furthermore, the Ba4 657 uid59173 strain has a greater number of proteins associated with sporulation processes within *C. botulinum* (n = 55). The comparative analysis algorithm used was CD-HIT, a tool for biological sequence clustering and comparison [68], which considers a percentage identity of ≥ 40.0% and where Kmer = 2 as the analysis criteria.

### Cytotoxicity and sporulation assays

Analyses of the cytotoxic effects and the sporulation capacities of the isolates followed the consensus conditions used previously for *C. difficile* [69,70]. First, the cryopreserved isolates were activated by streaking them onto brain heart infusion (Thermo Fisher Scientific) supplement medium (containing 3.7% BHI and 0.5% yeast extract) “BHIS”, and then grown for 24 h under anaerobic conditions. Using a sterile inoculating loop, the biomass from each isolate was transferred to 3 mL of BHIS supplemented with 0.1% (w/v) taurocholate and 0.2% (w/v) fructose [69], until an OD_600_ of 0.1 was reached. The cells were then incubated under the same anaerobic conditions, until they reached an OD_600_ of ≥ 0.5 (at ~ 48 hours). The OD_600_ was adjusted for isolates with higher ODs during the same incubation period.

The BHIS-cultured isolates were used for two purposes: 1) for sporulation assays, by plating 250 µL of each culture individually onto 35 ml of 70:30 sporulation medium [69] with subsequent incubation under anaerobic conditions over 5 days, which is the length of time identified as the best conditions for sporulation, and 2) for cytotoxicity tests, by recovering the supernatant from the remaining culture by centrifugation (4,000 rpm × 20 min), followed by an additional centrifugation step (13,000 rpm, 10 min) and two consecutive washes with sterile 1 × PBS. The washed supernatant was resuspended in 1 mL of saline solution and a 1:10 dilution was prepared. Aliquots (200 µL) of each supernatant concentration were transferred to 100 μl of 1 × 10^4^ Vero cells grown in 96-well plates as a confluent monolayer (previously trypsinized) in Dulbecco’s Modified Eagle’s Medium (DMEM, Sigma, St. Louis, MO, USA) containing bovine serum (0.5%). The cells were incubated for 16 h at 37°C in the presence of 5% CO2, and their viabilities were evaluated by a 3-(4,5- dimetiltiazol-2-ilo)-2,5-difeniltetrazol (MTT) assay, using dimethylsulfoxide to solubilize the crystals. Viability was calculated by comparing the results for each sample with a negative control, where the supernatant volume was replaced by saline solution.

Sporulation assays were conducted on the biomass recovered after incubation in 200 µL of a solution of Tween 80 in saline to allow for spore disaggregation, and each sample was subjected to mechanical disruption, by repeated pipetting and vortexing. The particles were flow cytometrically quantified, where particles of ≤0.3 µm in diameter were considered to be spores, and a granurality of 0.9 µm was considered to represent spore aggregates. The percentage sporulation efficiency was defined as the ratio of the number of spores with respect to the total number of particles recovered.

The ATCC BAA-1870 toxigenic reference strain, which contains *tcdA, tcdB* and *cdt* genes as confirmed by PCR, and Gcol.A112, a Colombian isolate identified in the *C. difficile* project in which the TCGs are missing, were included in the cytotoxicity and sporulation assays as controls.

## Results

### Clinical isolate establishment

The isolates were collected during the *C. difficile* infection (CDI) detection scheme that was conducted on stool samples from adult patients with diarrhea in Bogotá, Colombia. Two fecal samples identified by molecular screening [26] as CDI-positive and one as CDI-negative were obtained. These samples grew colonies on media that met the macro and microscopic selection criteria during verification by colony screening (VCS), as described in the methodology section; however, for each one, the microscopic appearance, shape and spore location were morphologically atypical (Supplementary Figure S1). We named these isolates Gcol.A2, Gcol.A11 and Gcol.A43, and conducted additional phenotypic screening on them. Despite the positivity for CDI by molecular tests, it was not possible to establish isolates from *C. difficile* or from any other *Clostridiales* species from the samples. The complete clinical information for the patients from whom the isolates were obtained is available in Supplementary Table S1.

### Preliminary phenotypical identification

All isolates shared the following traits: negative motility, negative urease activity and positive glucose and lactose fermentation. The exception was catalase activity, because Gcol.A43 was slightly positive for this trait unlike Gcol.A2 and Gcol.A11, which both lacked it. Because the catalase test is very useful for species discrimination, these results revealed that the Gcol.A2 and Gcol.A11 isolates did not belong to the *Bacillus* genus, thereby supporting the hypothesis that they were members of the *Clostridium* genus; however, the evidence was not sufficient to identify Gcol.A43 or to propose species designation for the other two isolates. Thereafter, the matrix-assisted laser desorption/ionization time-of-flight mass spectrometry (MALDI-TOF MS) results showed that the best match for Gcol.A11 corresponded to *C. paraputrificum* (score value: 2.261), whereas Gcol.A2 and Gcol.A43 matched *C. tertium* (score values: 2.216 and 2.102, respectively) (Supplementary Table S2).

### Taxonomic placement

Our preliminary analysis, based on *in silico* 16S RNA sequence extraction, provided support for Gcol.A11 belonging to *C. paraputrificum*, and Gcol.A2 and Gcol.A43 belonging to *C. tertium*, each with a percentage identity score of >98.0% (Supplementary Table S3). In addition, the SILVA Incremental Aligner (SINA) service, a tool included in the SILVA rRNA gene database [36], showed that both species belonged to the Clostridiaceae family (Supplementary Table S4).

We recovered 673 Clostridiales genomes from the PATRIC database [31,32], and we applied a quality control (QC) procedure to all the assemblies using an in house Sanger Perl script considering the parameters described in the materials and methods section. A total of 178 genomes were filtered out during the quality verification step (3.7% because the contig count failed and the remaining 96.3% because the 16S sequence was not found). Interestingly, the *C. paraputrificum* AGR2156 genome was one of the genomes filtered out. The results of this analysis were confirmed by verifying the quality of the different sequences for this strain that are publicly available in PATRIC [31,32], ENA [33] and NCBI [34] databases, to rule out possible biases related to different genome versions. All *C. paraputrificum* AGR2156 sequences were filtered out because the 16S sequence was missing.

A set of 497 genomes (including outgroups) was selected to conduct a preliminary verification step for grouping using *uprot*, a tool that identifies (by BLAST comparison), extracts, concatenates and aligns the sequences from 40 universal protein markers with single copy genes, previously used to generate high resolution phylogenies [38]. The amino acid sequences were retrieved after a rapid annotation step using Prodigal software [41]. The full list of molecular markers used for the high resolution phylogenetic reconstruction is described in the Supplementary Table S5. Three representative genomes from each of the seven Clostridiales families, which all agreed with the taxonomic descriptions available in the SINA Service, were selected for inclusion in the subsequent *uprot* analyzes [40], with the objective of maintaining a verification point for the grouping strategy. A single genome was used for the Heliobacteriaceae family, because it was the only one that passed the quality tests. With the Clostridiaceae family, 35 genomes corresponding to a genome representative of the total number of species found, plus all the genomes that grouped with the genomes analyzed, were included in the analyses. These parameters allowed us to select a definitive set of 65 genomes (Supplementary Table S6).

**Figure 1.**
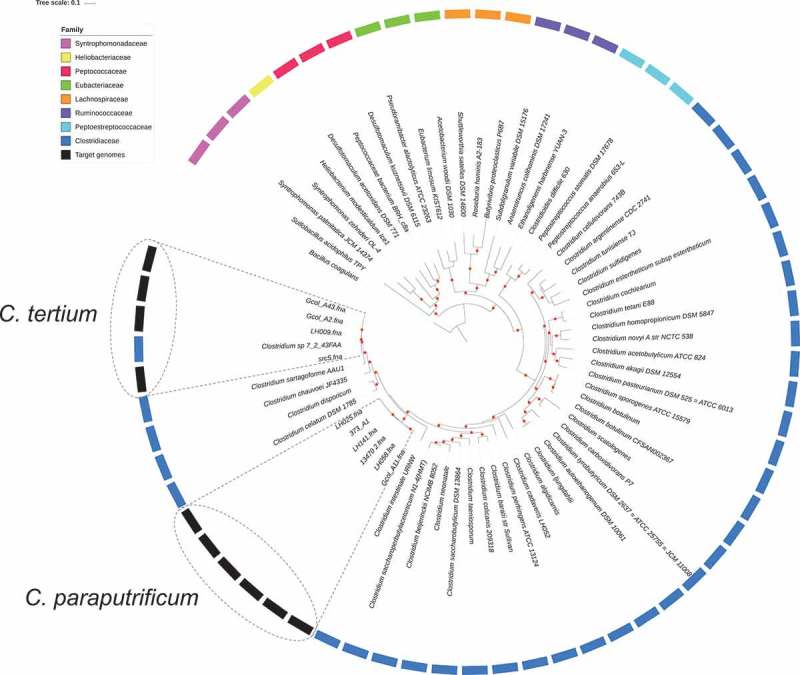
Phylogenetic relationships for Clostridiales species based of the concatenated sequence of 40 high-resolution molecular markers. a) Inter-species phylogenetic relationships for the selected genome dataset. A set of 65 Clostridiales genomes, belonging to eight families, was analyzed to propose the taxonomic designation of the target genomes (*C. tertium* and *C. paraputrificum*). Three representative genomes per family were included except for the Heliobacteriaceae family, where a single genome passed the preliminary quality tests, and for the Clostridiaceae family, for which a per-species genome was sought, because it was the family of interest. In the external ring, color was assigned to each Clostridiales family that was used in each analysis. The target genomes are marked with gray boxes, while boxes with dotted lines mark the grouping node for each target species. The *Bacillus coagulans* strain HM-08 (GCF_000876545.1) genome was included as outgroup. Red dots represent bootstrap values of ≥ 90.0.

The phylogenetic reconstruction based on the 40 high-resolution molecular markers’ alignment (Figure 1) showed a grouping for the Clostridiales family, according to the information found in the alignment obtained by SINA Service [71]. Comparing the phylogenetic reconstruction generated by *uprot* with the traditional classification method based on 16S rRNA (Supplementary Figure S2) allowed us to determine that the 40 universal markers method was able to discriminate a greater number of clusters and different members [40]. Further evaluation of the taxonomic assignments allowed us to determine that the species most closely related to the three isolates of interest were *C. celatum, C. disporicum, C. chauvoei* and *C. sartagoforme*.

### Genome descriptions and intra-taxa comparisons

The *C. paraputrificum* genome, obtained from the Gcol.A11 isolate, is 3.6 megabases (Mb) in length and potentially encodes over 3,500 proteins. The *C. tertium* genome obtained from Gcol.A2 is slightly larger (3.8–3.9 Mb), has more genes (> 3,700), and is similar to Gcol.A43, which contains 3,500 genes. The other characteristics of the isolates’ genomes and their gene annotations are shown in Table 1.

**Table 1.** Genome features of the *C. tertium* and *C. paraputrificum* clinical isolates.

	*C. tertium*		*C. paraputrificum*
Isolate name	Gcol.A2 (HCFO)	Gcol.A43 (CO)	Gcol.A11 (HCFO)
Total length (bp)	3,897,924	3,801,844	3,609,629
No. of contigs	60	55	91
N50 (bp)	281,242	178,423	98,983
G + C content (%)	28.12	29.05	30.27
No. of genes	3,743	3,591	3,569
CDS	3,585	3,440	3,408
tRNA	83	82	78
rRNA	13	11	11

HCFO, healthcare facility onset; CO, community onset; bp, base pairs; CDS, coding sequence

A dataset from the intra-taxa comparisons was selected during the process of taxonomical placement (Figure 1). The set contained five genomes for *C. tertium* and six genomes for *C. paraputrificum* (Table 2). Interestingly, the 72_43FAA genome was initially seen in databases containing *Clostridium* spp., but it consistently grouped with *C. tertium* genomes, so it remained in the analysis. A > 95.0 result was obtained for the average nucleotide identity (ANI) analysis [58], which allowed us to confirm that all the genomes within each group belonged to the same species (Supplementary Figure S3). Extended information of the genomes included in the groups used for the intra-taxa comparisons is available in Supplementary Table S7.

**Table 2.** Groups used for the intra-taxa comparisons.

Species	Number of genomes	Name	Assembly accession	Size (base pairs)	Source	Country	Reference
*C. tertium*	1	Gcol.A2	GCA_003284625.1	3,897,924	ICU	Colombia	This study
	2	Gcol.A43	GCA_003284645.1	3,801,844	Community	Colombia	This study
	3	LH009	GCA_900217175.1	3,970,462	29-week preterm infant	UK	[24]
	4	72_43FAA	GCA_000158375.2	3,827,748	Patient with Crohn’s disease	Canada	[99]
	5	src5	GCA_900205935.1	3,902,863	Bacteria isolated from the dairy production chain	UK	[100]
*C. paraputrificum*	1	Gcol.A11	GCA_003284655.1	3,609,629	ICU	Colombia	This study
	2	373_A1	GCF_001679805.1	3,488,595	***C. difficile*** intestinal infection	Chile	[25]
	3	13470_2	GCF_001404795.1	3,597,627	Healthy human donor	UK	[101]
	4	LH025	GCA_900217185.1	3,797,748	29-week preterm infant	UK	[24]
	5	LH058	GCA_900217195.1	3,776,795	32-week preterm infant	UK	[24]
	6	LH041	GCA_900217205.1	3,630,606	27-week preterm infant	UK	[24]

ICU, intensive care unit; UK, United Kingdom.

**Figure 2.**
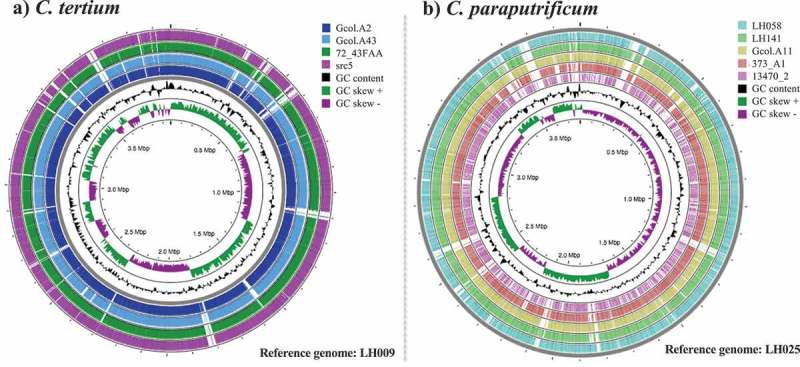
Intra-taxa comparisons of the complete genome sequences for (a) *C. tertium* and (b) *C. paraputrificum*. Analyses were developed in the CGview server [57]. The genome with the biggest size was selected as the reference for each species. Regions in white correspond to variable areas between genomes. Colors were assigned to the outer rings when sequences were found that each had a BLAST identity cutoff of 0.6, for the pairwise comparisons against the “reference genome”.

The GGskew (±), G + C content and preliminary BLAST comparisons on the complete genome sequences included in the intra-taxa comparisons are shown in Figure 2. These results provide the first indicator that the genomes differ from each other. The pairwise comparison analysis and the single nucleotide polymorphism (SNP) distance calculated for the data confirmed that, as a species, *C. paraputrificum* has the greatest number of genetic differences among the genomes (Supplementary Figure S4).

**Figure 3.**
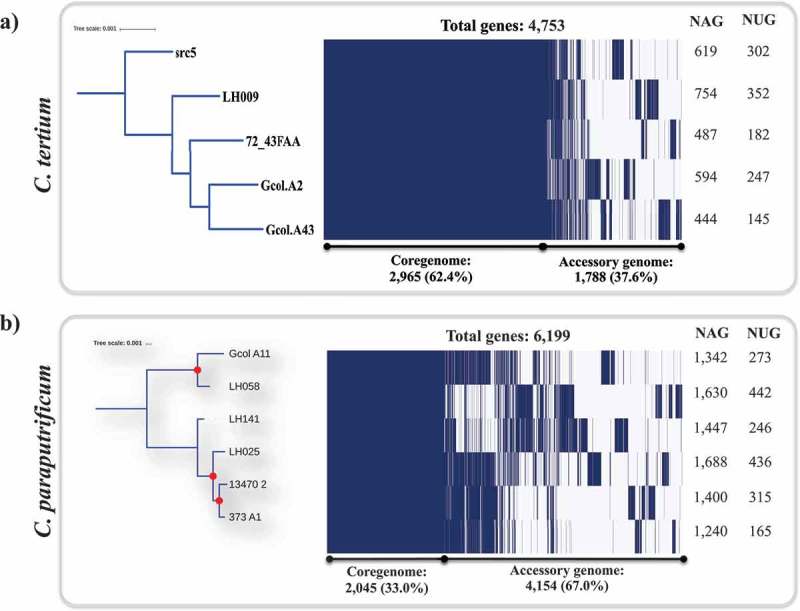
Pan-genome analyses for *C. tertium* (a) and *C. paraputrificum* (b) species as determined by Roary [61]. The parameters were defined as follows: Core genes: 99% ≤ strains ≤ 100% -accessory genes (15% ≤ strains < 95%). evolutionary insights between isolates based on the core genome (left panel). The core-genome tree generated was compared with a matrix where the core and accessory genes were either present or absent, which was graphically represented using the ipython *roary_plots.py* script. The top panel shows the color assignments for the different genes according to their lengths. NAG: number of accessory genes; NUG: number of unique genes. Red dots represent bootstrap values of ≥ 99.0.

The pan-genomes for *C. tertium* and *C. paraputrificum* were determined (Figure 3(a,b), respectively). In this analysis, the total number of *C. tertium* genes was 4,753, of which 37.6% (n: 1,788) corresponded to the accessory genome, unlike *C. paraputrificum*, where the total number of genes (n: 6,199) and the accessory genome (67.0%; n: 4,154) were greater. The central panel in Figure 3 is a schematic representation of the presence or absence of genes in the core and accessory genome . We also used the core genome from each species to phylogenetically construct the evolutionary history of the isolate sets for each species (Figure 3 left panel). Two clusters were identified for *C. paraputrificum*, and the most closely related isolates were again Gcol.A11 (the Colombian isolate) and LH058 (the isolate from a British preterm infant) in the first cluster, the latter of which clustered with other genomes from the same geographical origin (Figure 3(b)). The second cluster that contained *C. paraputrificum* included four other genomes, and the most related were two isolates from the American continent (373_A1 and 13470_2). The most relevant finding for *C. tertium* is that src5 (an **isolate of animal origin**) was identified as a potential ancestor in the analyzed dataset (Figure 3(a) left panel). The frequency of accessory genes determined from the pan-genome analyses is shown in Figure 3 (right panel). The LH025 *C. paraputrificum* genome from the pre-term infant isolates, contained the highest number of accessory genes (NAGs) and the highest number of unique genes (NUGs) (1,688 and 436, respectively), a result similar to that for LH058 with a NAG of 1,630 and a NUG of 442. In *C. tertium*, the NAGs and NUGs were lower than those in *C. paraputrificum*, whereas the isolate from the preterm infant displayed one of the highest values for both NAG (754) and NUG (352); these values are most similar to those from the animal isolate (NAG, 619, NUG, 302).

### Virulence factors identification

We sought to identify virulence factors such as toxin coding genes (TCGs), other virulence factors (OVFs), and antimicrobial resistance molecular markers (AMR-MMs) [72]. A total of 10 regions for *C. tertium* and 11 regions for *C. paraputrificum* were identified as “tox” region through our manual inspection (Supplementary Table S8, sheet 1 and 2, respectively). In the case of *C. tertium*, regions related with cholera toxin secretion domains (*epsF*_1 and *epsF*_2), toxin-antitoxin biofilm proteins (*tabA*_1 and *tabA*_2), toxic anion resistance protein (*telA*) and mRNA interferase EndoA (*ndoA*), were found in the five genomes analyzed. Four additional regions were exclusively present in single *C. tertium* genomes, one of these in Gcol_A2 (antitoxin HipB,transcriptional regulator, *hipB*), and the other three in src5 (Phage-related holin, Antitoxin *HicB* and HTH-type transcriptional regulator *immR*). For *C. paraputrificum* genomes the view of “tox” regions was more variable, being the transcriptional regulator *hipB*, cholera toxin secretion protein *epsF* and zeta toxin, the only regions present in all genomes. In general, these type of regions have been related with a high virulence in other pathogenic Clostridiales species as *C. difficile* [73].

Other TCGs were identified from short reads, these were: *toxA*, which is present in four of the six *C. paraputrificum* genomes analyzed (Gcol.A11, 373_A1, LH025 and LH141), which agrees with previous reports on this species [24]. A *toxA* gene (similar to *tcdA* encoding to Toxin A in *C. difficile*) was also identified in *C. tertium*, but only in the Colombian isolate (Gcol.A43). The third TCG identified was *toxB*, which only occurs in the Gcol.A11 genome and is reported here for the first time in *C. paraputrificum*. These last two toxins are widely recognized as the main toxins in *C. difficile*, and are described as causing the main clinical signs and symptoms in hosts affected by CDI [73]. Supplementary Figure S6(a–c) describe the phylogenetic reconstruction of three toxin coding genes (TCG) found in two Clostridiales species evaluated, where the sequences of the homologous genes found in other Clostridiales species were also included in the analysis.

Three OVFs were identified; the first was mRNA interferase (*endoA*), which is present in every *C. tertium* genome analyzed (Table 3). The gene associated with this molecular marker is involved in mRNA cleavage according to a previous analysis of specific sequences, and plays a role in secondary metabolite regulation in other bacteria such as *Bacillus subtilis* [74]. The *endoA* has already been reported in *C. difficile*, where it is described as a toxic component of the type II toxin-antitoxin system [75]. The second OVF, which was found in all the *C. paraputrificum* genomes except for Gcol.A11, but is present in the src5 *C. tertium* genome, is a phage-related holin, which in bacteria, particularly *C. difficile*, is involved in releasing the main bacterial toxins [76]. The third OVF we identified, which is present in both species genomes (13470_2 for *C. paraputrificum* and 72_43FAA for *C. tertium*) was the *nagH* hyaluronidase (a mu-toxin). This molecular marker is associated with a family of bacterial hyaluronidases that are involved in hyaluronate processing, and is produced by a number of pathogenic Gram-positive bacteria such as Group A *Streptococci* [77]. This protein has been recognized as a putative virulence factor in other Clostridiales species such as *C. perfringens*, in which has been proposed to act on connective tissue during gas gangrene [78].

**Table 3.** Virulence factors and antimicrobial resistance-related molecular markers identified across *C. tertium* and *C. paraputrificum* genomes.

			*C. tertium*					*C. paraputrificum*					
Type	Molecular marker	Access number	Gcol.A2*	Gcol.A43	7243FAA*	LH009*	src5	Gcol.A11	373_A1*	13470_2	LH025*	LH058*	LH141*
**TCGs**	*toxZ*. Zeta toxin. *Clostridium bornimense* replicon ^a)^	HG917868.1						M: 315T: 315 Q: 79 E: 1e-81 I: 80	M: 399T: 399 Q: 93 E: 8e-113 I: 77	M: 399T: 399 Q: 93 E: 8e-113 I: 77	M: 399T: 399 Q: 93 E: 8e-113 I: 78	M: 399T: 399 Q: 93 E: 8e-113 I: 79	M: 399T: 399 Q: 93 E: 8e-113 I: 77
	*toxA*. Gen encoding for toxin A in *Clostridium difficile* 630 ^c)^	YP_001087137		R:20; RL: 8,133; RBA: 289; PI: 92.73				R:134; RL: 8,133; RBA: 1,225; PI: 99.92	M: 86.0T: 225 Q: 11 E: 2e-15 I: 78		M: 87.8T: 202 Q: 19 E: 7e-16 I: 81		M: 44.6T: 44.6 Q: 1 E: 4e-04 I: 81
	*toxB*. Gen encoding for toxin B in *Clostridium difficile* 630 ^c)^	YP_001087135						R: 46; RL: 7,101; RBA: 583; PI: 99.66					\
**OVFs**	Phage-related holin in *Clostridium botulinum* strain B305 ^a)^	CP013850.1					M: 145 T: 145 Q: 89 E: 1e-33 I: 69		M: 266T: 266 Q: 85 E: 4e-67 I: 77	M: 313T: 313 Q: 85 E:3e-81 I: 80	M: 251T: 343 Q: 88 E:1e-651 I: 76	M: 37.4T: 108 Q: 11 E: 1.1 I: 86	M: 313T: 313 Q: 85 E:3e-81 I: 83
	mRNA interferase EndoA. PemK-like protein: Family of proteins mediating cell death through inhibiting protein synthesis through the cleavage of single-stranded RNA. Reference: *Clostridium pasteurianum* BC1 ^a)^	CP013019.1	M: 329T: 329 Q: 94 E: 3e-90 I: 81	M: 316T: 316 Q: 88 E: 2e-86 I: 82	M: 316T: 316 Q: 88 E: 2e-86 I: 82	M: 316T: 316 Q: 91 E: 2e-86 I: 82	M: 69.8T: 69.8 Q: 23 E: 5e-12 I: 79	M: 274T: 284 Q: 88 E: 1e-69 I: 83					
	*nagH*. Hyaluronidase mu-toxin in *Clostridium perfringens* str. 13 ^b)^	BA000016.3			L: 314C: 81.21 I: 81					L: 204C: 83.33 I: 83			
	*tetA*(P)_1. Tetracycline-resistance genes of *Clostridium perfringens* ^b)^	AB054980			L: 1,263; C: 100 I: 92.08								
	*tetB*(P)_3. Conjugation and replication regions of the tetracycline resistance plasmid pCW3 from *Clostridium perfringen ^b)^*	NC_010937			L: 1,959; C: 100 I: 98.52								
**AMR-MMs**	*Staphylococcus aureus* 23S rRNA with mutation that confers resistance to linezolid ^d)^	NZ_CP009828.1	R: 38; RL: 2,926; RBA: 567; PI: 91.89	R: 9,462 RL: 2,926; RBA: 567; PI: 91.89			R: 906 RL: 2,926; RBA: 465; PI: 93.12			R: 3,044 RL: 2,926; RBA: 567; PI: 91.36			
	*Streptococcus pneumoniae* 23S rRNA mutation conferring resistance to macrolides and streptogramins antibiotics ^d)^	NZ_CP018138.1	R: 34 RL: 2,926; RBA: 555; PI: 91.73	R: 8,656 RL: 2,904; RBA: 243; PI: 90.12									
	*Clostridium difficile* EF-Tu (mutations confer resistance to elfamycin) ^d)^	NC_017174.1						R: 60 RL: 1,194; RBA: 1,194; PI: 98.99					
	*cdeA*. Multidrug efflux transporter with antiporter function. Confers resistance to fluoroquinolones in *E. coli* and acriflavin in *C.difficile* ^d)^	AJ574887.1						R: 34 RL: 1,326; RBA: 342; PI: 100					
	*gyrA.C*onfers resistance to fluoroquinolones in *Clostridium* difficile ^d) e)^	NC_009089.1						R: 42 RL: 2,427; RBA: 345; PI: 99.13					
	FOLP_20. Sulfonamide-resistant dihydropteroate synthases; group:FOLP ^e)^	AM180355.1 (gene1650)						R: 12 RL: 804; RBA: 195; PI: 100					
	RPOB_3. Rifampin-resistant beta-subunit of RNA polymerase RpoB; group:RPOB ^e)^	AM180355.1 (gene120)						R: 102 RL: 3,717 RBA: 1,718 PI: 100					
	TUFAB_4. Elfamycins; mechanism:EF-Tu inhibition; group:TUFAB ^e)^	NC_017174_1						R: 60 RL: 1,194 RBA: 1,194 PI: 98.99					

Toxin coding genes (TCGs), other virulence factors (OVFs), and antimicrobial resistance molecular markers (AMR-MMs) [72], determined by: a) manual search; b) ABRicate; from c) to e) using Ariba, being c) Vfdb; d) Card; e) Megares database. The results identified using “Manual search” were based on BLAST comparison: M: max score; T: total score; Q: query cover (%); E: E value; I: Identity (%). For the ABRIcate results, we have reported: L: length (nt); C: coverage (%) and I: Identity (%). The Ariba results are described with the following data: R: reads (#); RL: reference lengh (nt); RBA: reference base assembled (nt); PI: percentage of identity (%). * Genomes whose fastq files were not publicly available and where it was not possible to develop the Ariba analysis. Empty wells indicate where results are lacking.

Eight AMR-MMs were identified in *C. paraputrificum*, with most of them occurring exclusively in Gcol.A11 (7/8) (Table 3). These markers are associated with resistance to macrolides, streptogramins, elfamycin, fluoroquinolones, acriflavin, sulfonamides and rifampin. One additional marker identified in 13470_2 is associated with linezolid resistance. The AMR-MMs identified in *C. tertium* (Table 3), albeit in smaller numbers than in *C. paraputrificum* (n = 2), are associated with resistance to linezolid, macrolides and streptogramins. Only two genomes carried these markers: Gcol.A43 (two markers) and src5 (one encoding linezolid resistance).

### Sporulation process-related proteins

**Figure 4.**
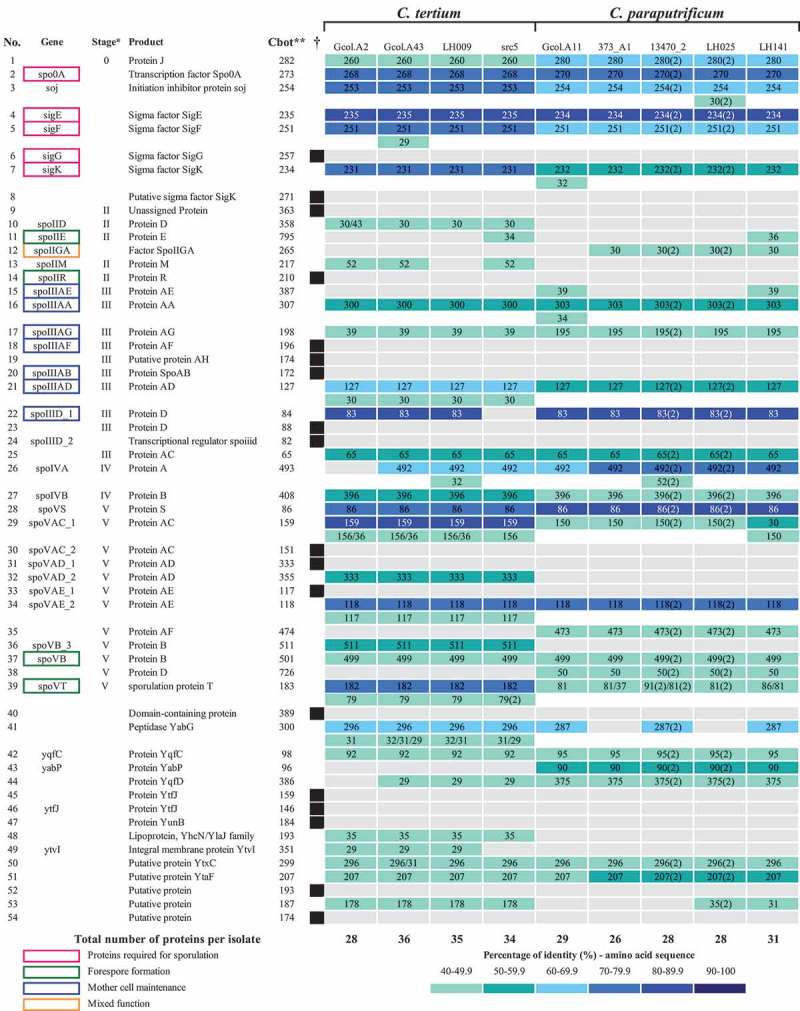
Identification of the proteins involved in sporulation processes for each analyzed species. As reference the reported proteins in *C. botulinum* Ba4 657 uid59173 were used as comparisons in the CD-HIT suite, taking the following parameters into account: % identity > 40 and Kmer = 2. * Stage: sporulation pathway step based on the reported information. ** *Cbot: C. botulinum* Ba4 657 uid59173; Numbers inside each well indicate the reference genome length and the numbers in parenthesis represent the number of copies found. Parenthetical numbers indicate the number of times proteins were identified more than once in the multifasta files that were used for each analysis. † Black boxes represent the proteins reported (in the reference genome), that are missing in all the evaluated isolates. Because no sporulation proteins were identified in 72_43FAA (*C. tertium*) and LH058 (*C. paraputrificum*) they were excluded from the analyses. The proteins are listed according to their timing of action in the sporulation pathways, in agreement with previous reports [78,98].

Of the 55 proteins involved in the sporulation process reported in the *Cbot* Ba4 657 uid59173 reference genome, an average of 28 (range, 26–31) protein homologs were identified in 5/6 *C. paraputrificum* genomes and in 34 (range, 28–36) of the 4/5 *C. tertium* genomes evaluated here under the parameters described in the Methods section (Figure 4). None of the proteins related to the sporulation process were found in 72_43FAA (*C. tertium*) and LH058 (*C. paraputrificum*). Of the set of 18 proteins known to be involved in the sporulation process, none were found in the genomes analyzed, including the SigG sigma sporulation factor, which is one of the main regulators required at the start of the sporulation process [78]. However, other sporulation-related proteins were found.

We identified the amino acid sequences from sporulation process-related proteins, most of which had sizes similar to those reported for the reference sequences, with sequence identity percentages of mostly 40–70%, the exceptions being nine proteins that shared up to 90% identity with each other. A high level of genetic variation was found in genomes 13470_2 and LH025, which contained a high frequency of complete multi-copy proteins where more than one sequence was identified in the multifasta files from the assemblies; interestingly, the additional copies found in the Colombian isolates appeared to be truncated and had variable sequence identity ranges, especially for Gcol.A43 (Figure 4).

### Cytotoxicity and sporulation efficiency

**Figure 5.**
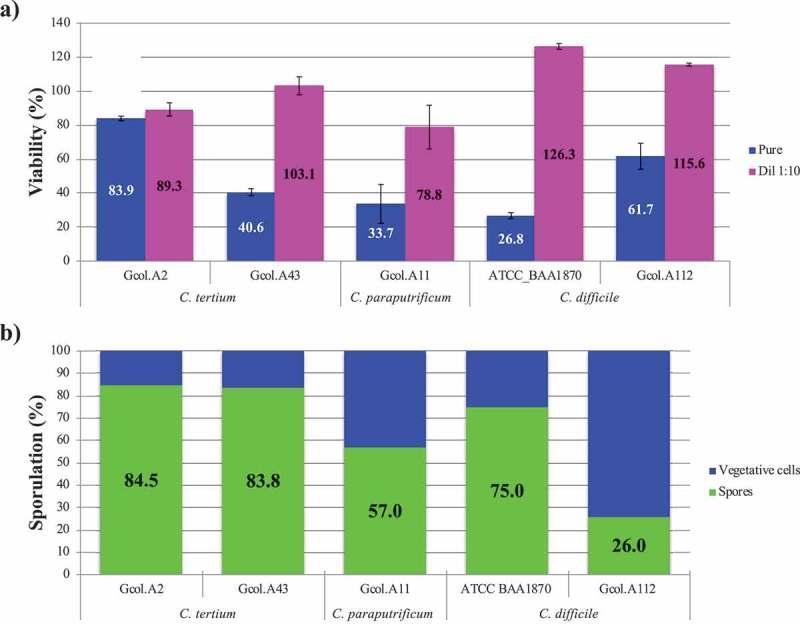
Experimental validation of the cytotoxic effect (a) and the sporulation capacity (b) of each Colombian isolate. *C. difficile* isolates considered to be toxigenic (ATCC_BAA1870) and non-toxigenic (Gcol.A112) were included as controls. The percentage viability (%), as measured by the MTT assay, is expressed in comparison with the negative control, where DMEM medium was used to replace the volume of the isolate with 50% (v/v) saline solution, after which all the samples received the same experimental treatment. Each isolate supernatant was evaluated directly and as a 1:10 solution. The sporulation percentage, as measured by flow cytometry, was based on the size (≤0.3 μM) and granularity (≤0.9 μM). The percentage sporulation was calculated by considering the number of spores with respect to the total number of particles analysed.

Cytotoxicity testing of the Vero cells showed that *C. paraputrificum* and *C. tertium* both displayed cytotoxicity (Figure 5(a)). With *C. paraputrificum*, the viability percentage for the Gcol.A11 isolate was similar to that for the toxigenic ATCC_BAA1870 *C. difficile* strain, which was used as a positive control. With *C. tertium*, the viability percentage differed between the two Colombian isolates, with a greater reduction seen in Gcol.A43 than in Gcol A2. The reduction in cell viability for Gcol A2 was less than 20%, causing an even lower effect than that observed for the *C. difficile* isolate, in which no toxin coding sequences were identified.

Regarding the spore-producing ability of the isolates (Figure 5(b)), *C. tertium* displayed a greater sporulation efficiency compared with *C. paraputrificum* (57.0%), with percentages of at least 83% for the two *C. tertium* isolates; these values are even higher than those for the two *C. difficile* control isolates.

## Discussion

Herein, three new isolates identified were subjected to genome-wide sequencing to determine their taxonomic designation and phylogenetic relationships using a phylogenomic approach. This method was able to overcome the resolution limitations that have been reported for traditional 16S rRNA-based methods, where mis designation and unreliable assignments can occur [79]. Oher approaches have been used to obtain better intra-taxa phylogenetic relationships within clostridiales species, such as increasing the number of 16S rRNA targets [2] and whole genome sequence data analysis [79,80], but no consensus for the classification strategy has been reached so far.

With this background in mind, automated software linked to the most widely used tools for bacterial taxonogenomics [38,40] was used in the present study to discern the phylogenomic signatures of clostridiales species. An approach based on multiple markers has proven useful for reconstructing highly resolved trees [40], and has also been used to infer the taxonomy of other bacterial groups, such as the Chlamydiales order [81]. The phylogenetic tree generated (Figure 1(a)) showed family clusters within the clostridiales order. In this tree, the two species of interest belong to the Clostridiaceae family, which includes pathogens that are hazardous to public health [3], and are closely related to unfamiliar species (e.g., *C. celatum, C. disporicum, C. chauvoei*, and *C. sartagoforme*) previously recognized as only being pathogenic to non-human animals until less than 10 years ago when they began to be associated with aggressive clinical diseases in humans [82–84]. A comparison of the findings based on the traditional 16S rRNA approach (Supplementary Figure S2) showed that the multiple marker method produced the best resolution. Then, we propose it as an alternative for studying complete taxonomy within an order in the future.

The characteristics of the isolated species at the genome level (Table 2) showed that the values are close to the mean value for the full genome size range for *Clostridium* species, which for the *C. cellulosi* DG5 strain is 2,229,578 bp, whereas that of the *C. saccharoperbutylacetonicum* N1-4 (HMT) strain is up to 6,666.445 bp [32]. The sizes are concordant with those described in the only report on the complete genomes for these species [24], being slightly larger in the genomes isolated from the pre-term infant (Table 1). The intra-taxa comparisons revealed that the genomes we analyzed have highly variable zones (Figure 2), a feature that is more apparent in *C. paraputrificum*. This is concordant with the pan-genome results (Figure 3(b)), where it was found that the pan-genome contains 6,199 genes and that 67.0% of it corresponds to the accessory genome. Hence, we propose that *C. paraputrificum* exhibits high diversity at the genome level. The genome diversity levels for *C. tertium* were lower, but highly variable areas were also identified (Figure 2) and 34.2% of the 4,571 genes that are part of the pan-genome are accessory genes (Figure 3(a)). Our intra-taxa phylogenetic reconstructions on *C. paraputrificum* revealed the existence of two clusters in the 40 molecular marker and core-genome phylogeny analyses (Figure 3(b)). Interestingly, despite the genome from the pre-term infant’s isolate (LH025) being larger than that from the Colombian isolate from the adult ICU patient (Gcol.A11), both isolates were genetically closely related. A notable finding from the phylogenetic reconstruction on *C. tertium* was the observation that src5 was the most probable ancestor within the analyzed dataset (Figure 3(b)).

These findings have important implications for the evolutionary histories of these species, because they indicate the possibility of genome plasticity in bacteria, as opposed to the concept of clonality [85], but they are supported by the principles of the origin of diversity in prokaryotic species [86], which in turn is in agreement with what was previously reported for the most studied pathogenic species of *Clostridiales* [87]. Evidence to support the proposal of genome plasticity in clostridiales species is as follows: i) the large genome size compared with other species (with the genome sizes of the species under analysis being very close to the *C. difficile* value of 4,293,049 bp in the *C. difficile* 630Δerm strain), a trait associated with gene acquisition as an adaptive strategy for exploiting multiple adverse environments [87,88]; ii) the locations of the variable regions among the genomes at specific sites (Figure 2 and Supplementary Figure S5), which further supports the multipartite genome organization hypothesis in which gene acquisition occurs in specific genome regions, generating organization in the form of fragments that become indicators of selection-driven evolution [89]; and iii) the presence of a high percentage of accessory genes (Figure 3), known as the adaptive genome, which represents a low level of conservation in the genomes and is linked to reduced periods of adaptation of the species to its host, thereby determining its capacity for virulence and antibiotic resistance [90]. Unfortunately, in this study we were not able to conduct MIC testing (an important limitation of this study) on the studies isolates to determine the true expression AMR loci. Future studies must consider this premise to reach more approachable conclusions. These findings, together with the differences found between the genomes from each species present in certain locations as hotspots, suggests that some genes may have been laterally transferred, as reported previously in other human pathogens [12], including *C. difficile* [91].

The pathogenic effects of *Clostridium* species are associated with their toxin-producing abilities, but these are quite variable effects between species, as has been reported for the two species of interest where the existence of chitinases [22] and *toxA* [24] in *C. paraputrificum*, and sialidases in *C. tertium*, together with toxA only in the Colombian isolate genome, were discovered [20]. However, the virulence factor descriptions available to this study (Table 3) allowed us to identify coding sequences for three different TCGs (*toxZ, toxA* and *toxB*). For the case of *C. paraputrificum*, i) the existence of genes in this bacterium that may code for other mechanisms possibly involved in the action of toxins (*toxZ* in all the isolates which belong to a large family of clostridial dual enzymes associated with pathogenic effects in other clostridiales species (Table 3)). ii) Homologous genes to *tcdA* and *tcdB* that showed pathogenic features in other clostridiales species and iii) the experimental verification of the cytotoxic effects of *C. paraputrificum* on Vero cells (Figure 5(a)), support the hypothesis that *C. paraputrificum* is a Clostridiales species potentially pathogenic [14]. Unfortunately, the biological function of *toxZ* is not currently known, which added to the fact that some genes associated with toxins have been found to be involved in processes of metabolic regulation for certain microbial species. It is not possible to clarify the role of this gene in the pathogenic potential of the herein studied species. Therefore, future studies are needed to elucidate its regulatory mechanisms and biological activity. These findings are of special interest since despite the absence of information about the frequency of infection in humans and/or animals, their presence and corresponding expression of these genes coding for toxins could cause a negative impact on the health status of infected individuals (as septicemia and liver abscess), when they find the right environment for their proliferation (as is the case of old age or immunosuppression), as has been reported in the few case reports available for humans [14,92], or even in healthy young patients [93].

In contrast, this is the first study to identify the *toxA* gene in *C. tertium*, a species previously reported to be non-toxigenic. This gene was identified in the genome from one of the Colombian isolates (Gcol.A43), which possibly also induced the highest cytotoxic effect on Vero cells (Figure 5(a)), and likely provides important information about its pathogenic potential in humans. Evaluation of OVFs showed that src5 carries a gene encoding a phage-related holin potentially involved in toxin secretion/phage lysis holing. Although we identified sequences that potently encode for virulence factors in both analyzed species (*C. paraputrificum* and *C. tertium*). The limited number of isolates analyzed and the lack of knowledge about their evolutionary histories does not allow us to decipher possible associations between the genetic organization and the impact over their hosts. Then, the process of characterizing the potential pathogenic effects of these species should be further studied.

Another important factor in the life cycle of clostridiales species is their ability to produce spores [78]. From this analysis, *C. paraputrificum* and *C. tertium* were found to have most of the genes known to be indispensable for the sporulation processes (Figure 4), with the number of such genes being the greatest in *C. tertium*. The sporulation capacities of these Colombian isolates under culture conditions revealed that they had a high efficiency of spore production and a high level of sigma factors (Figure 4(b)), possibly indicating that these species may be more amenable than others to surviving adverse conditions. Hence, these species have the signaling pathways required to resist adverse conditions, which in the clinical context is recognized as being important for infection dissemination [94]. For *C. tertium* in particular, its highly efficient spore production might even indicate a strong environmental adaptability [95]. In this way, the production of spores is recognized as a virulence factor, the flagship of the Clostridiales species, which allows them to remain viable in adverse environments [78], but which become a challenge for the clinical management of infected patients, since they facilitate their dissemination and persistence. However, this has only been extensively analyzed in widely studied species, such as *C. difficile* [96] and *C. perfringens* [97], but the impact of sporulation on other species such as those in this study is still unknown.

In conclusion, by using the phylogenomic approach reported herein we were able to type three isolates recovered fortuitously during CDI detection screening as belonging to the Clostridiaceae family. The highly detailed whole genome analyses we conducted on them and other database-deposited clostridiales species revealed high levels of intra-taxa diversity, accessory genomes containing TCGs, OVFs and AMR-MMs (especially in *C. paraputrificum*), with proteins involved in sporulation processes more highly represented in *C. tertium*. Crucially, verification of the high cytotoxic effect of *C. paraputrificum* and the high spore-forming capacity of *C. tertium*, leads us to identify important virulence factors and for that to propose that the two species are important from a public health perspective because of their potential pathogenic effects on their hosts (particularly in *C. paraputrificum*). Thus, further studies involving expanded datasets of foreseen Clostridiales species are warranted for generate high quality *Clostridium* genomes, identifying sporulation genes and virulence factors, and building a comprehensive phylogeny. It is necessary to conduct comprehensive studies inferring the origin of these genes, the gain/loss in specific lineages, and the possible links to the clinical manifestations observed for the different species/strains. Nonetheless, this study provides basic knowledge about the virulence and antibiotic-resistance factors in *C. tertium* and *C. paraputrificum*, as well as providing new insights into the biology of these species.

## Data Availability

The whole genome data that support the findings of this study are openly available DDBJ/ENA/GenBank under the accession PRJNA472349. The accession numbers for the assembled genome version described in this paper are: QGMV00000000 for *Clostridium tertium* Gcol.A2, QGMU00000000 for *Clostridium tertium* Gcol.A43 and QGMT00000000 for *Clostridium paraputrificum* for Gcol.A11.

## Supplementary material

The supplemental data for this article can be accessed here.
