# Supplementary material for: Comparative genomics identifies potential virulence factors in Clostridium tertium and C. paraputrificum
Source: Virulence. 2019 Jul 13;10(1):657–76. doi: 10.1080/21505594.2019.1637699 (PMC6629180; doi:10.1080/21505594.2019.1637699)

Supplementary Fig. S1. Microscopic appearance of clinical isolates analyzed in comparison with the *C. difficile* reference strain ATCC BAA-1870. a) Gram stained. b) spore morphology verification using malachite green staining with subsequent contrast of the vegetative cells safranin.

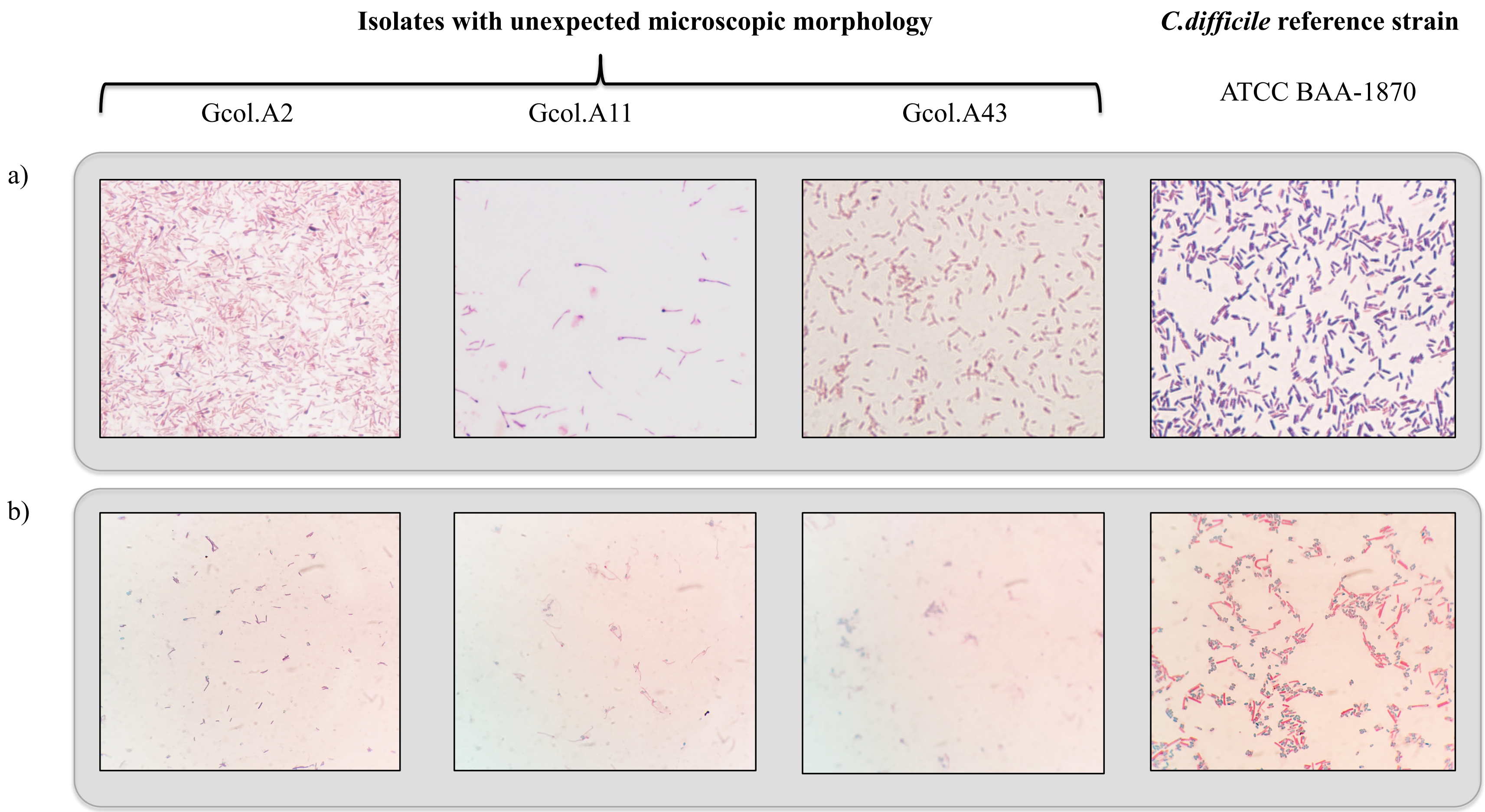

Supplement: Supplemental Material [file kvir-10-01-1637699-s001.zip › 1. Supplementary Fig. S1.pdf]
