# Supplementary material for: Comparative genomics identifies potential virulence factors in Clostridium tertium and C. paraputrificum
Source: Virulence. 2019 Jul 13;10(1):657–76. doi: 10.1080/21505594.2019.1637699 (PMC6629180; doi:10.1080/21505594.2019.1637699)
Supplement: Supplemental Material [file kvir-10-01-1637699-s001.zip › 11. Supplementary Fig. S4.pdf]

Supplementary Figure S4. Pairwise comparison analysis beetwen genomes included in the analyses per each species. Comparisons were developed Circoletto, an online visualization tool based on Circos (Darzentas, 2010), using blastn with -F F -e 1e-10 -E -1 -v 200 -b 200. Ribbons were created from 1,000 local alignments, then were coloured by bitscore and depth-ordered by score, highest-scoring at the top using 'score/max' ratio (occording with color scale). Information under each pairwise comparison shows the number of local alignments (LA) and the minimun and maximus bitscore in parentesis. SNP distance (calculated using NUCmer (NUCleotide MUMmer) version 3.1) is shown at right of each comparison (in blue).

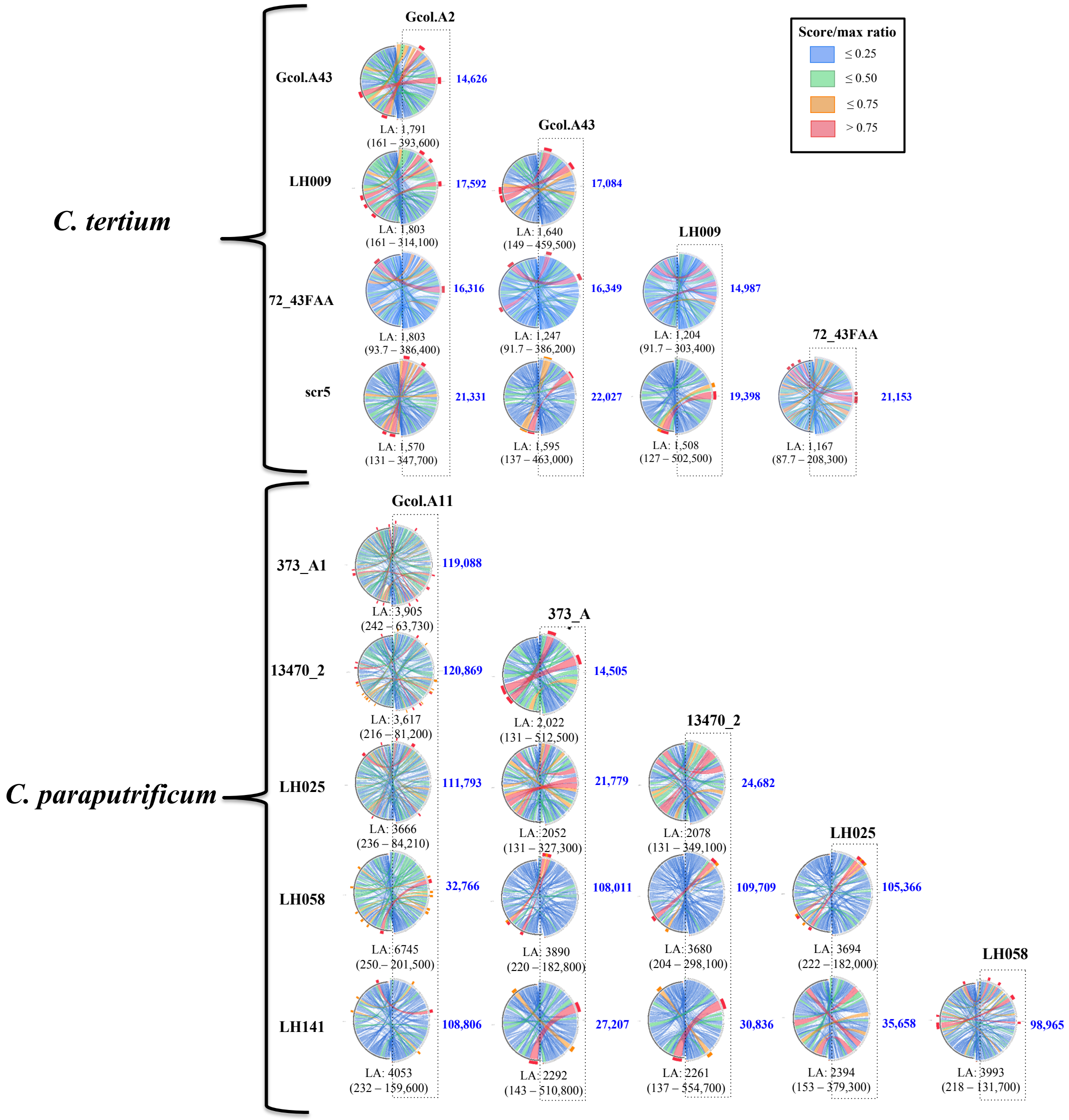

References

Darzentas, N. (2010). Circoletto: visualizing sequence similarity with Circos. *Bioinformatics* 26, 2620-2621. doi: 10.1093/bioinformatics/btq484
