# Supplementary material for: Comparative genomics identifies potential virulence factors in Clostridium tertium and C. paraputrificum
Source: Virulence. 2019 Jul 13;10(1):657–76. doi: 10.1080/21505594.2019.1637699 (PMC6629180; doi:10.1080/21505594.2019.1637699)
Supplement: Supplemental Material [file kvir-10-01-1637699-s001.zip › 2. Supplementary Table S1.pdf]

Supplementary Table S1. Clinical information about patients from healthcare facility-onset (HCFO).

	**Gcol.A2**	**Gcol.A11**
Concomitant diseases:	Congestive heart failure	Dysentery - Chronic diarrheal disease
Period of time:	33 days in HCFO, 19 of these in intensive care unit	61 days in HCFO, 31 of these in intensive care unit
History of multiple antibiotic use:	Albendazole, Ivermectin and Ertapenem	Meropenem, Metronidazole and Teclozan
State at the end of the hospitalization period:	Live	Dead

* Both patients were ≥80 years old
