# Supplementary material for: Comparative genomics identifies potential virulence factors in Clostridium tertium and C. paraputrificum
Source: Virulence. 2019 Jul 13;10(1):657–76. doi: 10.1080/21505594.2019.1637699 (PMC6629180; doi:10.1080/21505594.2019.1637699)

Supplementary Figure 2 (Fig. S2). Phylogenetic relationships inferred from 16S rRNA sequences analysis of the definitive dataset. Red dots represent bootstrap values of ≥ 90.0.

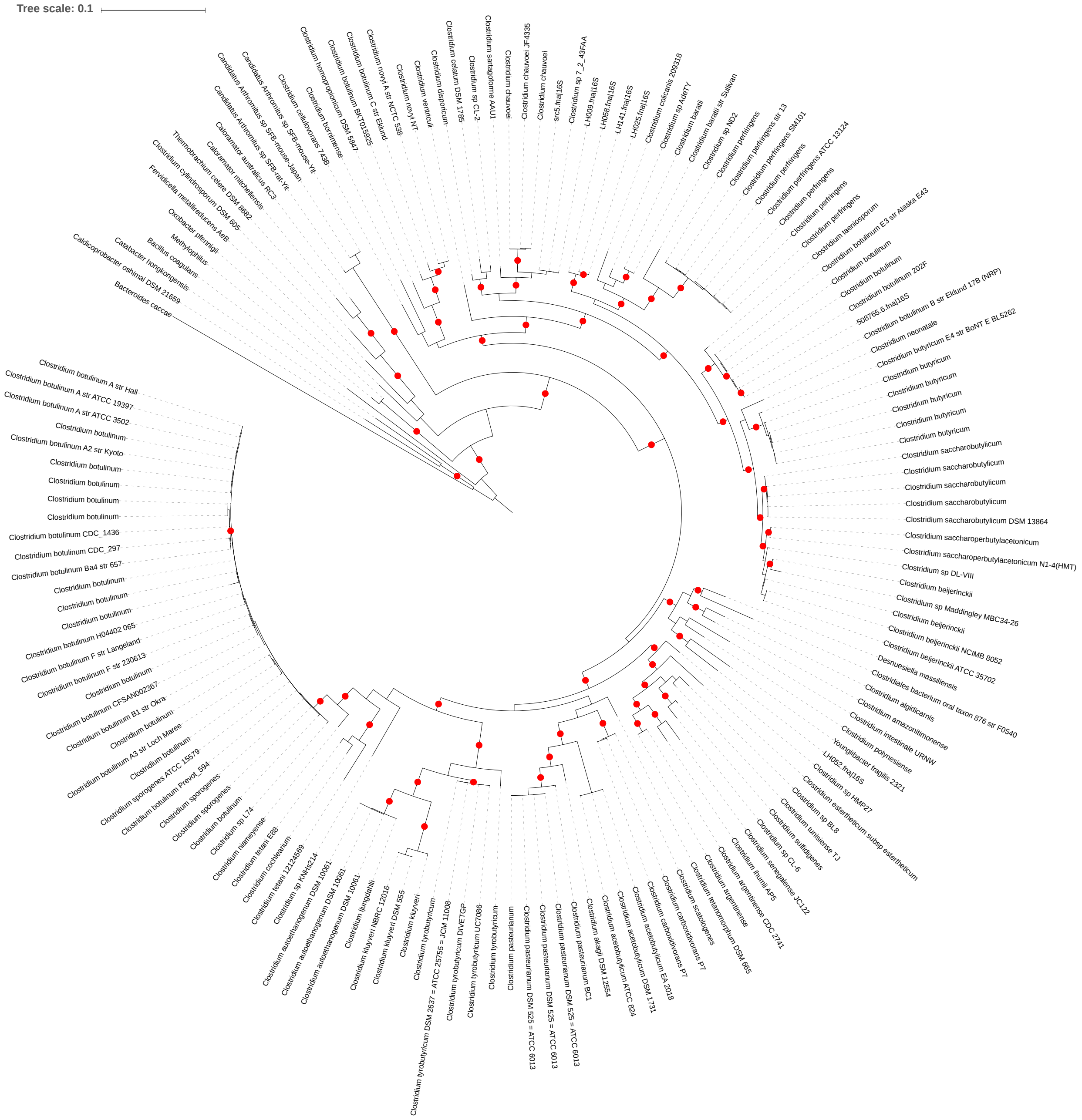

Supplement: Supplemental Material [file kvir-10-01-1637699-s001.zip › 8. Supplementary Fig. S2.pdf]
