# Supplementary figures and images for: Comparative genomics identifies potential virulence factors in Clostridium tertium and C. paraputrificum
Source: Virulence. 2019 Jul 13;10(1):657–76. doi: 10.1080/21505594.2019.1637699 (PMC6629180; doi:10.1080/21505594.2019.1637699)

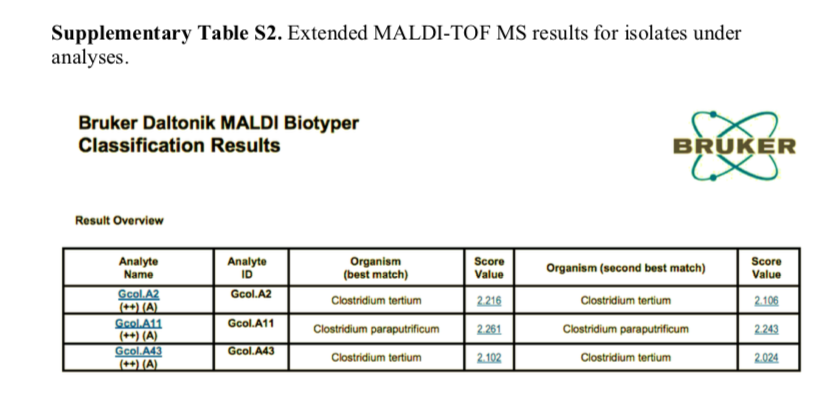

Supplement: Supplemental Material [file kvir-10-01-1637699-s001.zip › 3. Supplementary Table S2.png]

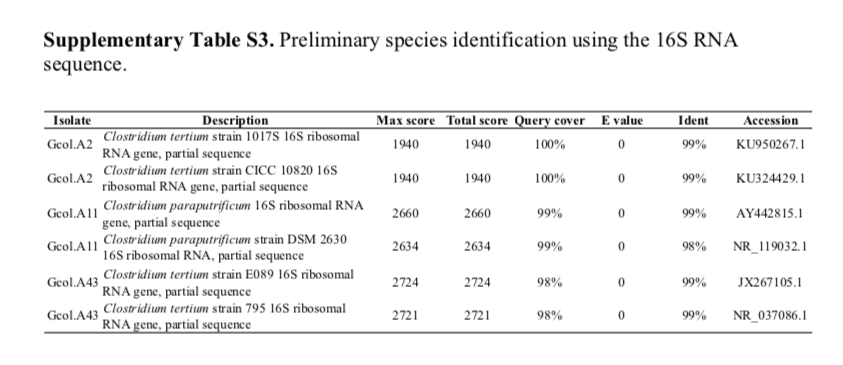

Supplement: Supplemental Material [file kvir-10-01-1637699-s001.zip › 4. Supplementary Table S3.png]

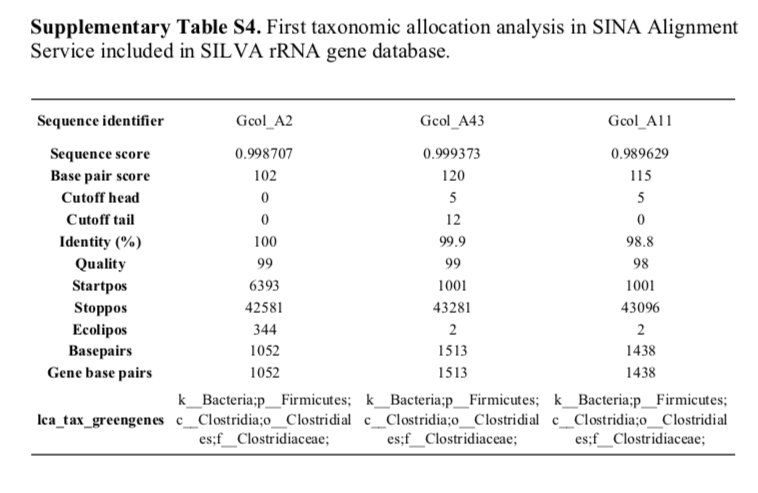

Supplement: Supplemental Material [file kvir-10-01-1637699-s001.zip › 5. Supplementary Table S4.png]

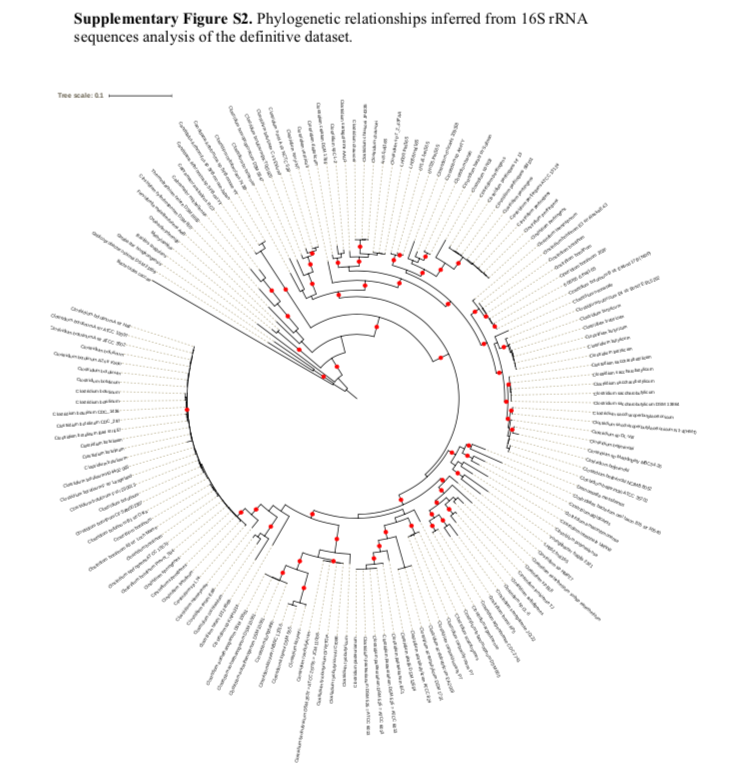

Supplement: Supplemental Material [file kvir-10-01-1637699-s001.zip › 8. Supplementary Fig. S2.png]

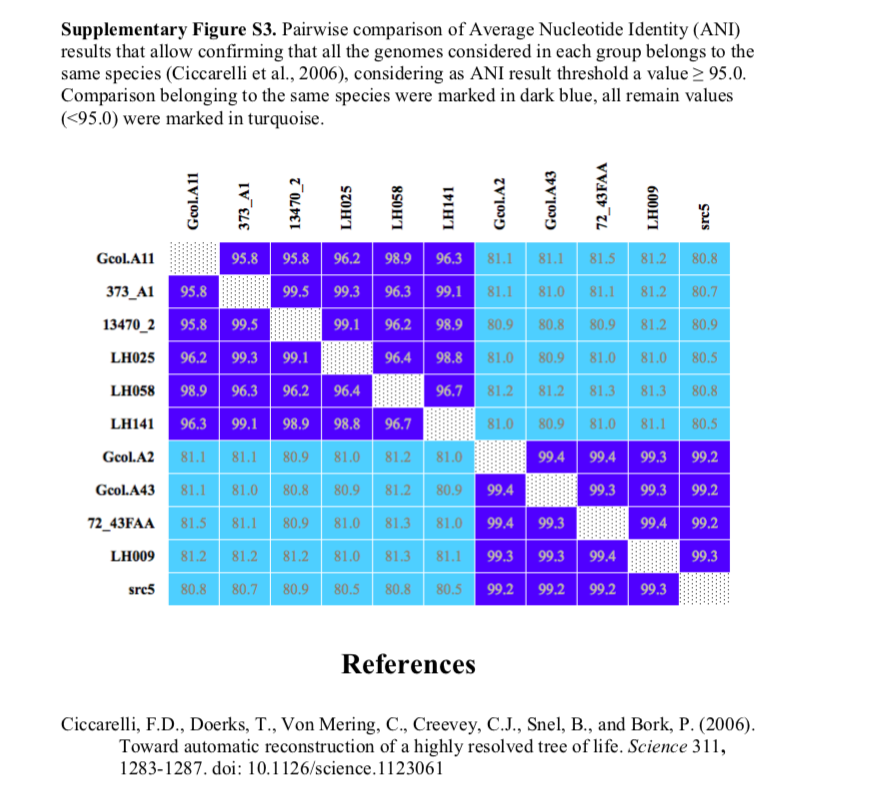

Supplement: Supplemental Material [file kvir-10-01-1637699-s001.zip › 9. Supplementary Fig. S3.png]
